# Supplementary figures and images for: Effect of quitting immediately vs progressively on smoking cessation for smokers at emergency department in Hong Kong: A posteriori analysis of a randomized controlled trial
Source: PLoS One. 2023 Jan 26;18(1):e0280925. doi: 10.1371/journal.pone.0280925 (PMC9879435; doi:10.1371/journal.pone.0280925)

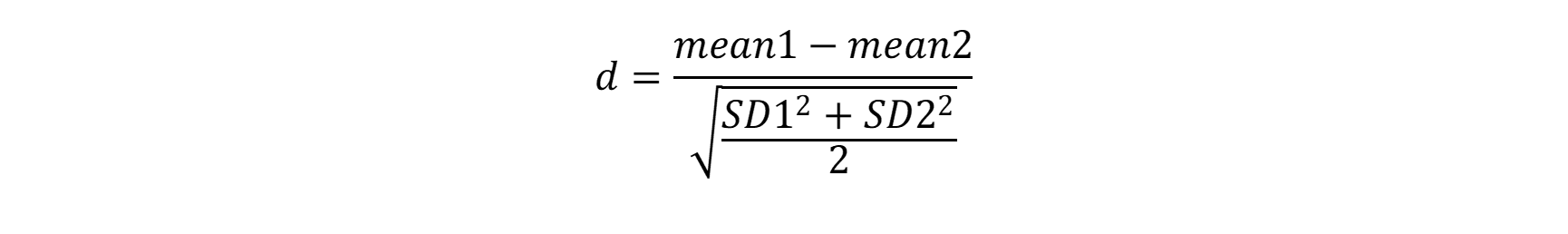

Supplement: S1 Fig — (TIF) [file pone.0280925.s002.tif]

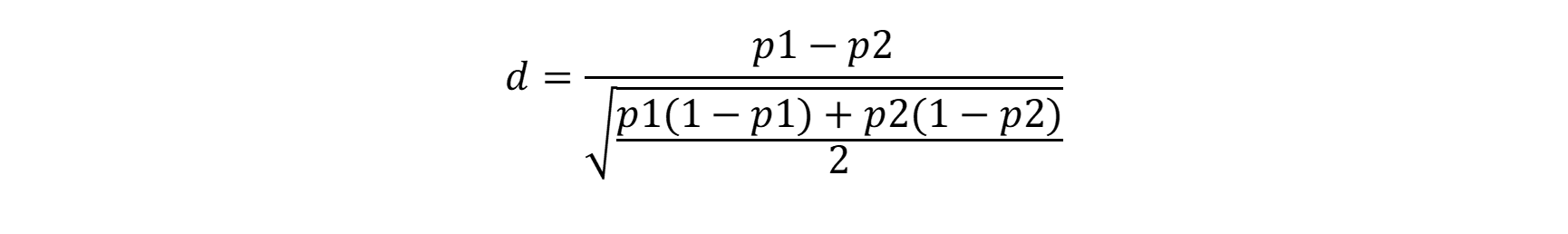

Supplement: S2 Fig — (TIF) [file pone.0280925.s003.tif]
