# Supplementary material for: Effect of quitting immediately vs progressively on smoking cessation for smokers at emergency department in Hong Kong: A posteriori analysis of a randomized controlled trial
Source: PLoS One. 2023 Jan 26;18(1):e0280925. doi: 10.1371/journal.pone.0280925 (PMC9879435; doi:10.1371/journal.pone.0280925)
Supplement: S1 Table — (DOCX) [file pone.0280925.s004.docx]

S1_Table. Comparison of baseline characteristics and smoking profiles among subjects in the QP group and control group in the original unmatched sample and the propensity-score matched sample

|  | Propensity-score matched sample | | | |
| --- | --- | --- | --- | --- |
|  | QP  (n=545) | Control  (n=545) | P value | Standardized differences |
| Age, mean(SD), y | 47.7(15.2) | 47.7(16.9) | 0.961 | 0.000 |
| Gender |  |  | 0.556 | 0.029 |
| Male | 484(88.8) | 490(89.9) |  |  |
| Female | 61(11.2) | 55(10.1) |  |  |
| Marital status |  |  | 0.291 | 0.053 |
| Single/Separate/Widowed | 221(40.6) | 204(37.4) |  |  |
| Married/Cohabit | 324(59.4) | 341(62.6) |  |  |
| Employment status |  |  | 0.336 | 0.048 |
| Unemployed/Retired | 137(25.1) | 151(27.7) |  |  |
| Employed | 408(74.9) | 394(72.3) |  |  |
| Educational level |  |  | 0.683 | 0.018 |
| Tertiary | 27(5.0) | 30(5.5) |  |  |
| Secondary or below | 518(95.0) | 515(94.5) |  |  |
| Monthly household income, US $ |  |  | 0.661 | 0.021 |
| ≥3825 (HKD 30000) | 73(13.4) | 78(14.3) |  |  |
| <3825 (HKD 29999) | 472(86.6) | 467(85.7) |  |  |
| Smoking-related chronic disease |  |  | 0.807 | 0.013 |
| Yes | 35(6.4) | 37(6.8) |  |  |
| No | 510(93.6) | 508(93.2) |  |  |
| Health utility score by SF-6D^a^ | 0.57(0.1) | 0.57(0.1) | 0.702 | 0.000 |
| Daily cigarette consumption | 15.0(7.9) | 14.9(7.6) | 0.860 | 0.013 |
| Nicotine dependence by Heaviness of Smoking Index (HIS)^b^ |  |  | 0.670 | 0.021 |
| Moderate to heavy(3-6) | 303(55.6) | 296(54.3) |  |  |
| Light(≤2) | 242(44.4) | 249(45.7) |  |  |
| Age at starting smoking weekly | 17.2(5.7) | 17.6(7.0) | 0.303 | 0.067 |
| Tried to quit smoking for more than 24 hours |  |  | 0.898 | 0.007 |
| Yes | 357(65.5) | 359(65.9) |  |  |
| No | 188(34.5) | 186(34.1) |  |  |
| Tried to reduce smoking for more than 24 hours |  |  | 0.671 | 0.021 |
| Yes | 268(49.2) | 261(47.9) |  |  |
| No | 277(50.8) | 284(52.1) |  |  |
| Readiness to quit |  |  | 0.628 | 0.036 |
| Quit ≤ 30 days | 133(24.7) | 145(26.6) |  |  |
| Quit ≥ 30 days | 405(75.3) | 400(73.4) |  |  |
| Self-efficacy against tobacco by SEQ-12^c^ | 29.1(10.8) | 28.3(11.5) | 0.251 | 0.069 |

Continuous variables are reported as mean ± standard deviation. Dichotomous variables are reported as N (Percent).

Abbreviations: SF-6D, Shot-Form Six-Dimension; SEQ-12, Smoking Self-Efficacy Questionnaire.

^a^The SF-6D is composed of 6 multilevel dimensions. The SF-6D scores were weighted from a sample of the general population, which ranged from 0 to 1.

^b^The Heaviness of Smoking Index, a 2-item index from multiple-choice response options (0-3), was determined by assessing cigarettes smoked per day and latency to smoke after waking; the higher the indexes, the greater smoking nicotine dependence.

^c^On a 12-item 5-point Likert-type scale in the SEQ-12, responses ranged from “not at all sure” to “absolutely sure.” A summary score of the SEQ-12 ranged from 12 to 60, with higher scores indicating higher self-efficacy
