# Supplementary material for: Effect of quitting immediately vs progressively on smoking cessation for smokers at emergency department in Hong Kong: A posteriori analysis of a randomized controlled trial
Source: PLoS One. 2023 Jan 26;18(1):e0280925. doi: 10.1371/journal.pone.0280925 (PMC9879435; doi:10.1371/journal.pone.0280925)
Supplement: S2 Table — (DOCX) [file pone.0280925.s005.docx]

S2_Table. Cessation outcomes of subjects in the QP group vs. control group

|  | Original unmatched sample | | | |  | Propensity-score matched sample | | | | |  |
| --- | --- | --- | --- | --- | --- | --- | --- | --- | --- | --- | --- |
|  | QP group (n=545) | control group (n=784) | P value | Crude ORs^b^ in the unmatched sample |  | QP group (n=545) | control group (n=545) | P value | Crude ORs^b^ in the matched sample | Adjusted ORs^c^ in the matched sample | |
| Biochemically validated abstinence | | | | | | | | | | | |
| 6 months | 19(3.5) | 22(2.8) | 0.481 | 1.25(0.67, 2.34) |  | 19(3.5) | 4 (0.9) | 0.006 | 3.90(1.45, 10.52) | 3.96(1.42, 11.06) | |
| 12 months | 24(4.4) | 33(4.2) | 0.863 | 1.05(0.61, 1.80) |  | 24(4.4) | 10 (1.8) | 0.015 | 2.46(1.17, 5.20) | 2.88(1.31, 6.33) | |
| Self-reported 7-day point prevalence of abstinence | | | | | | | | | | | |
| 6 months | 43(7.9) | 73(9.3) | 0.367 | 0.83(0.56, 1.24) |  | 43(7.9) | 25 (4.6) | 0.024 | 1.78(1.07, 2.96) | 1.91(1.14, 3.21) | |
| 12 months | 48(8.8) | 67(8.5) | 0.868 | 1.03(0.70, 1.52) |  | 48(8.8) | 27(5.0) | 0.012 | 1.85(1.14, 3.02) | 2.07(1.24, 3.44) | |
| Self-reported reduction of ≥ 50% in cigarette consumption^a^ | | | | | | | | | | | |
| 6 months | 89(17.7) | 127(17.9) | 0.952 | 0.99(0.74, 1.34) |  | 89(17.7) | 76 (14.6) | 0.176 | 1.26(0.90, 1.76) | 1.26(0.89, 1.77) | |
| 12 months | 96(19.3) | 105(14.6) | 0.031 | 1.40(1.03, 1.89) |  | 96(19.3) | 69 (13.3) | 0.010 | 1.56(1.11, 2.18) | 1.59(1.12, 2.26) | |

Subjects lost to follow-up were assumed to be active smokers with no changes in their habit.

^a^ The quitters were excluded in both numerators and denominators.

^b^ Crude ORs= Crude Odds Ratios. Crude estimates from the univariable logistic regression

^c^ Adjusted ORs= Adjusted Odds Ratios. Adjusted estimates from the Generalized Estimating Equations model adjusted for age, marital status employment status, income, health utility score, daily cigarette consumption, nicotine dependence level, and readiness to quit, the smoking self-efficacy at the baseline and the random effect of hospitals.
