# Supplementary material for: Effect of quitting immediately vs progressively on smoking cessation for smokers at emergency department in Hong Kong: A posteriori analysis of a randomized controlled trial
Source: PLoS One. 2023 Jan 26;18(1):e0280925. doi: 10.1371/journal.pone.0280925 (PMC9879435; doi:10.1371/journal.pone.0280925)
Supplement: Attachment — Submitted filename: Responses to the comments-2.docx [file pone.0280925.s007.docx]

Dear Editor,

Re: "Effect of Quitting Immediately vs Progressively on Smoking Cessation for Smokers at Emergency Department in Hong Kong: A Posteriori Analysis of a Randomized Controlled Trial" (PONE-D-21-03778)

We sincerely thank the editor and reviewer’s very useful and constructive comments. We have made changes accordingly. Please also refer to the following point-by-point responses to the comments from the reviewer and editor.

Please kindly let me know in case any responses are not clear or the information is not adequate to clarify concerns. Thanks for giving us an opportunity to revise the manuscript.

Please also note that all data are belong to the funder (Health and Medical Research Fund, Food and Health Bureau, Hong Kong Special Administrative Region). The data can be accessed only with the permission by the Bureau (<https://rfs2.fhb.gov.hk/english/welcome/welcome.html>.)

Sincerely,

Authors

**Response to the Journal requirements**

| Comment 1 | 1. Please ensure that your manuscript meets PLOS ONE's style requirements, including those for file naming. The PLOS ONE style templates can be found at https://journals.plos.org/plosone/s/file?id= wjVg/ PLOSOne_formatting_sample_ main_body.pdf and <https://journals.plos>. org/ plosone/s/file?id=ba62/PLOSOne_formatting_sample_title_authors_ affiliations.pdf |
| --- | --- |
| Response 1 | We have changed the format of the manuscript and ensured that the manuscript meets the requirement of PLOS ONE. |
| Comment 2 | 2. We note that the grant information you provided in the ‘Funding Information’ and ‘Financial Disclosure’ sections do not match.  When you resubmit, please ensure that you provide the correct grant numbers for the awards you received for your study in the ‘Funding Information’ section. |
| Response 2 | Thank you for pointing out the inconsistent information. We have corrected the Funding information in the manuscript (Page 23, line 13). |
| Comment 3 | 3. We note that you have indicated that data from this study are available upon request. PLOS only allows data to be available upon request if there are legal or ethical restrictions on sharing data publicly. For more information on unacceptable data access restrictions, please see http://journals.plos.org/plosone/s/data-availability#loc-unacceptable-data-access-restrictions.  In your revised cover letter, please address the following prompts:  a) If there are ethical or legal restrictions on sharing a de-identified data set, please explain them in detail (e.g., data contain potentially sensitive information, data are owned by a third-party organization, etc.) and who has imposed them (e.g., an ethics committee). Please also provide contact information for a data access committee, ethics committee, or other institutional body to which data requests may be sent.  b) If there are no restrictions, please upload the minimal anonymized data set necessary to replicate your study findings as either Supporting Information files or to a stable, public repository and provide us with the relevant URLs, DOIs, or accession numbers. For a list of acceptable repositories, please see http://journals.plos.org/plosone/s/data-availability#loc-recommended-repositories.  We will update your Data Availability statement on your behalf to reflect the information you provide. |
| Response 3 | Thanks for the comment. We have contacted with the Funder and then update our Data Availability statement as below:  All data are belong to the funder (Health and Medical Research Fund, Food and Health Bureau, Hong Kong Special Administrative Region). The data can be accessed only with the permission by the Bureau (<https://rfs2.fhb.gov.hk/english/welcome/welcome.html>.) |
| Comment 4 | 4. Please review your reference list to ensure that it is complete and correct. If you have cited papers that have been retracted, please include the rationale for doing so in the manuscript text, or remove these references and replace them with relevant current references. Any changes to the reference list should be mentioned in the rebuttal letter that accompanies your revised manuscript. If you need to cite a retracted article, indicate the article’s retracted status in the References list and also include a citation and full reference for the retraction notice. |
| Response 4 | We have checked the reference list and ensure that it is complete and correct. |

**Response to comments from Reviewer**

| Comment 1 | 1- Tables 1 to 3: For each baseline characteristic, provide only one p-value for comparing all three groups. If the p-value is significant, perform step-down tests to determine pairwise differences. |
| --- | --- |
| Response 1 | Following the reviewer’s suggestion, the one-way ANOVA analysis was used to compare the differences of baseline variables among the three groups, and then a Tukey's honestly significant difference (HSD) post hoc test was performed to specific groups difference for the variables showing significant difference in the one-way ANOVA analysis. Please find the results in the table 1 to 3. The description of the statistical analysis method was revised accordingly as below (Page 9, line 8):  “For the variables showing significant difference in ANOVA, the Tukey's honestly significant difference post-hoc test the Games -Howell post-hoc test and were performed when the assumption of equal variances was met and not met, respectively.”  In addition, we will keep the standardized differences in table 1 and table 2 as they may allow the readers to well understand that demographic and smoking profiles among the three groups were well balanced after the PSM. |
| Comment 2 | 2-Page 9: Explain the rationale for using a GEE model when adjusting for baseline characteristics rather than applying multivariate logistic regression models. |
| Response 2 | We are apologized for the mistake of the description. Actually we used a Generalized Logistic Mixed Model (GLMM) to calculate the odds ratios as the data in this study were collected in multiple research center and the GLMM allowed us to adjust the random effect of the hospitals in the model. We have corrected the description in the manuscript as below (Page 9, line 20) and the notes under the table 4 accordingly:  “A Generalized Logistic Mixed Model (GLMM) was then used to calculate the adjusted odds ratios (aORs) for primary and secondary outcomes after adjusting for characteristics at baseline and the random effect of hospitals using the matched sample.” |
| Comment 3 | 3- Page 10, Line 1: The figure number had been omitted. |
| Response 3 | Thank you for pointing out the mistake, we have revised it as “Figure 1” in Page 10, line 1. |
| Comment 4 | 4- Provide a post-hoc power calculation. |
| Response 4 | Thank you for your suggestion, the results of power calculations have been added in the manuscript, please find it in table 5 and page 18, lines 1-4 as below:  “The observed power (1-β) of quitting immediately and quitting progressively on the biochemically validated quit rate, the self-reported quit rate, and self-reported reduction of cigarette consumption were then calculated using G*power.” |
| Comment 5 | 5- Explain the graphical results in figure 2. |
| Response 5 | The explanation of the results in figure 2 has been added in the results section as below (Page 19, line 1-5):  “The scatterplot and ﬁtted line analysis showed that the values of both the absolute and percent cigarette reduction at the 1-, 3-, and 6-month follow-ups were associated with the biochemically validated abstinence as assessed at the 12-month follow-up (Fig 2). The R2 showed that the percent cigarette reduction (a-2, b-2, c-2) could better predict the 12-month abstinence than the absolute cigarette reduction (a-1, b-1, c-1).” |

Additional comments from Editor

| Comment | We've checked your submission and before we can proceed, we need you to address the following issues:  1. We note that your Data Availability Statement reads: "Please also note that all data are belong to the funder (Health and Medical Research Fund, Food and Health Bureau, Hong Kong Special Administrative Region). The data can be accessed only with the permission by the Bureau (<https://rfs2.fhb.gov.hk/english/welcome/welcome.html>.)"  Since your data is from a third-party, please address the following:  1. Please explain how others can access these datasets and confirm that others would be able to access these data in the same manner as the authors.  2. Please also confirm that the authors did not have any special access privileges that others would not have. |
| --- | --- |
| Response | Thanks for the questions. For question (1), we have added the information to and amend the “Data Availability Statement” as below:  “All data are belong to the funder (Health and Medical Research Fund, Food and Health Bureau, Hong Kong Special Administrative Region). The data can be accessed only with the permission by the Bureau (https://rfs2.fhb.gov.hk/english/welcome/welcome.html). However, the corresponding author (principal investigator and research grant recipient) will, upon reasonable request by others, contact the Bureau for approval to access the datasets.  For question (2), I would like to confirm that the corresponding author (principal investigator and research grant recipient) can access the datasets. However, he should inform the Bureau in advance for approval if any data will be publicized. I also confirm that the authors did not have any special access privileges that others would not have. |
