# Supplementary material for: Effect of quitting immediately vs progressively on smoking cessation for smokers at emergency department in Hong Kong: A posteriori analysis of a randomized controlled trial
Source: PLoS One. 2023 Jan 26;18(1):e0280925. doi: 10.1371/journal.pone.0280925 (PMC9879435; doi:10.1371/journal.pone.0280925)
Supplement: Attachment — Submitted filename: Responses to Reviewers.docx [file pone.0280925.s008.docx]

Dear Editor,

Re: "Effect of Quitting Immediately vs Progressively on Smoking Cessation for Smokers at Emergency Department in Hong Kong: A Posteriori Analysis of a Randomized Controlled Trial" (PONE-D-21-03778R1)

We sincerely thank the editor and reviewer’s very useful and constructive comments. We have made changes accordingly. Please also refer to the following point-by-point responses to the comments from the reviewer and editor.

Please kindly let me know in case any responses are not clear or the information is not adequate to clarify concerns. Thanks for giving us an opportunity to revise the manuscript.

Please also note that all data are belong to the funder (Health and Medical Research Fund, Food and Health Bureau, Hong Kong Special Administrative Region). The data can be accessed only with the permission by the Bureau (<https://rfs2.fhb.gov.hk/english/welcome/welcome.html>.)

Sincerely,

Authors

**Response to comments from Reviewer**

| Comment 1 | Minor Revision required:  Include the full details of the power calculation. The power calculation should include: sample size, alpha level (indicating one or two-sided), minimal detectable difference (plus the standard deviation when appropriate) and statistical testing method. |
| --- | --- |
| Response 1 | Thanks for the reviewer’s suggestion. We have added the following information related to sample size calculation in the text:  “The sample size was calculated according to a previous trial [3] of a smoking reduction plus nicotine replacement therapy intervention involving 1154 Chinese adult smokers unwilling to quit smoking (biochemically validated quit rate of 4.4% [10 of 226] in the control group and 8.0% [74 of 928] in the intervention group at 6months). To detect a two-sided significant difference between groups with a power of 80% and significance level of 5%, the required sample size was estimated to be 1088 participants (544 in each group). Given an expected attrition rate of approximately 30% at the 6-month follow-up, the target was at least 1554 individuals (777 in each group). Between July 4, 2015 and March 17, 2017, 1571 smokers who presented at 4 major emergency departments consented to participate in this randomized controlled trial and were randomized into an intervention group (n = 787) and a control group (n = 784).” |
